# Enhancing Hemophilia Care: Real-World Outcomes Following Switching to Extended Half-Life Factor VIII in Greece—The TOOL Study

**DOI:** 10.3390/life16060881

**Published:** 2026-05-25

**Authors:** Helen Pergantou, Sofia Vakalopoulou, Efrosyni Nomikou, Marina Economou, Anna Kouramba, Aikaterini Michalopoulou, Athina Dettoraki, Eleni Moka, Alkistis Adramerina, Olga Katsarou

**Affiliations:** 1 Haemophilia Centre, Haemostasis and Thrombosis Unit, Aghia Sophia Children’s Hospital of Athens, Thivon 1 and Papadiamantopoulou, Goudi, 11527 Athens, Greece; 2 Haemophilia Centre of Northern Greece, 2nd Propedeutic Department of Internal Medicine, Aristotle University of Thessaloniki, 54636 Thessaloniki, Greece; 3 Blood Tranfusion Unit, Haemophilia Centre, Ippokration General Hospital of Athens, 11527 Athens, Greece; 4 1st Pediatric Department, School of Medicine, Faculty of Health Sciences, Aristotle University of Thessaloniki, 54636 Thessaloniki, Greeceaadrame@auth.gr (A.A.); 5 Blood Transfusion Unit, National Reference Centre for Congenital Bleeding Disorders, Laiko General Hospital of Athens, 11527 Athens, Greece

**Keywords:** hemophilia, efmoroctocog alfa, switch, bleeding, joint health, prophylaxis

## Abstract

**Introduction**: Extended half-life (EHL) factor VIII (FVIII) products aim to reduce treatment burden and improve bleeding control in hemophilia A. Real-world evidence remains essential to complement clinical trials. **Aim**: To evaluate clinical outcomes following switching from standard half-life (SHL) rFVIII to efmoroctocog alfa in routine clinical practice in Greece. **Methods**: Multicenter observational pre–poststudy including patients with moderate to severe hemophilia A. Outcomes were assessed during the 12 months before and after switching. The primary endpoint was change in annualized bleeding rate (ABR). Secondary endpoints included annualized joint bleeding rate (AjBR), infusion frequency, joint health, pain, and FVIII consumption. **Results**: Sixty patients were included. Following switching, ABR decreased from 6.8 to 3.2 (53%), and AjBR from 6.4 to 2.9 (55%), *p* < 0.001. Reductions were more pronounced in patients switching from on-demand treatment, while more modest improvements were observed among patients already on prophylaxis. HJHS significantly decreased from 17.9 to 11.5 (*p* < 0.007), accompanied by a decrease in pain scores (*p* < 0.001), in available paired subsets. Weekly infusion frequency decreased (3.2 to 2.2; *p* < 0.001), while mean dose per infusion increased, resulting in no consistent reduction in total annual FVIII consumption. No inhibitor or treatment-related adverse events have been observed. **Conclusions**: Switching to efmoroctocog alfa in routine practice was associated with improved bleeding outcomes, reduced infusion frequency, and better joint-related parameters. These findings support the real world feasibility and clinical utility οf EHL FVIII therapy, while further controlled studies are needed to better define the independent effect of product switching from changes in treatment regimen and other potential confounders.

## 1. Introduction

Hemophilia A (HA) is an X-linked recessive bleeding disorder, primarily affecting males, resulting from a deficiency or absence of coagulation factor VIII (FVIII). Globally, approximately 142,000 individuals are diagnosed with HA, including an estimated 33,000 cases within the European Union across diverse racial and ethnic groups. In Greece, the number of affected individuals ranges between 800 and 850, depending on clotting factor activity levels [1].

Hemophilia classification is based on endogenous factor activity: severe (<1%), moderate (1–5%), and mild (>5–40%) [2,3]. Severe HA significantly increases the risk of spontaneous and traumatic internal bleeding in joints, muscles, and organs, leading to recurrent hemorrhages and heightened morbidity, particularly due to arthropathy [4,5].

Treatment objectives focus on clinical outcomes such as annualized bleeding rate (ABR), annualized joint bleeding rate (AjBR), pain associated with arthropathy, hematoma count, and range of motion loss. Moreover, health-related quality of life (HRQoL) is increasingly recognized as pivotal in contemporary hemophilia management and should be considered when implementing treatment strategies [2,3].

Two principal therapeutic approaches exist: on-demand therapy, where FVIII is administered during bleeding episodes, and prophylactic FVIII therapy. While on-demand treatment effectively manages acute bleeding, it does not prevent arthropathy [5]. Prophylactic FVIII treatment, conversely, reduces bleeding episodes, limits hospitalizations, and enhances long-term joint function [6].

Prophylactic FVIII therapy usage is rising, yet the short half-life of recombinant FVIII (rFVIII) (approximately 6–12 h) necessitates frequent infusions (three to four times weekly) to maintain trough levels above 1–3 IU/dL (1–3%). The requirement for multiple intravenous infusions imposes a considerable treatment burden, often resulting in reduced compliance or patient refusal, and poses additional challenges related to venous access, especially in children, sometimes necessitating central venous devices that carry risks of infection and thrombosis [7].

Advancements in bioactive clotting factors with prolonged circulation have led to extended half-life (EHL) and higher FVIII and IX trough levels in plasma [8]. This progress reduces infusion frequency, potentially improving adherence to prophylaxis, and may encourage episodic patients to transition to prophylaxis [9]. Efmoroctocog alfa, a recombinant FVIII Fc fusion protein (rFVIIIFc), has demonstrated sustained efficacy in acute bleeding, perioperative management, and routine prevention in males previously treated for severe hemophilia [4].

Prophylaxis is considered optimal where economically feasible and is recommended to commence early, prior to bleeding events. Its benefits include substantial reductions in bleeding episodes, improved joint condition, and enhanced quality of life (HRQoL) [10,11]. EHL agents facilitate less frequent injections and higher trough levels, promoting greater physical activity and improved HRQoL [12,13]. Higher trough levels may also help prevent subclinical bleeding and consequent joint disease, especially in vulnerable patients [14]. Prophylaxis is similarly endorsed for adult patients with hemophilia who have undergone on-demand therapy. Transitioning to prophylaxis results in significant reductions in ABR compared to episodic therapy, with rates decreasing by up to 76% [4], which, in turn, reduces hospitalizations and resource utilization.

This study aims to evaluate clinical outcomes observed after switching patients with moderate to severe hemophilia A from SHL FVIII to efmoroctocog alfa in routine clinical practice.

Importantly, the study population included patients previously treated either on-demand or with prophylaxis, representing distinct scenarios that may have influenced outcomes. Therefore, the findings should be interpreted as real-world observations rather than evidence of comparative efficacy or superiority.

## 2. Materials and Methods

### 2.1. Study Design and Setting

The TOOL study was a multicenter, observational, non-interventional cohort study with both retrospective and prospective components, designed to evaluate the real-world effectiveness of switching from standard half-life (SHL) recombinant factor VIII (rFVIII) to extended half-life (EHL) rFVIIIFc (efmoroctocog alfa) in patients with hemophilia A.

The study included a retrospective period of 12 months prior to treatment switch and a prospective follow-up period of 12 months (±1 month) after switching. Both retrospective and prospective data included bleeding outcomes, treatment exposure, hospitalization, joint health assessments, and patient-reported outcomes (PROs).

The study was conducted in five hemophilia treatment centers in Greece (Athens and Thessaloniki), including both pediatric and adult units, ensuring representation across age groups and disease severity.

### 2.2. Study Population

Patients were eligible if they met the following criteria:

- Male patients with a confirmed diagnosis of moderate or severe hemophilia A (FVIII ≤ 5%);
- Previously treated with SHL rFVIII, either on-demand or prophylactically;
- Switched to efmoroctocog alfa as part of routine clinical practice;
- Availability of clinical data for at least 12 months prior to treatment switch;
- Provided written informed consent.

Patients were excluded if they:

- Had a high-titer FVIII inhibitor at baseline;
- Were participating in an interventional clinical trial.

Patients with a history of inhibitors or low-titer inhibitors were eligible and monitored throughout the study.

### 2.3. Treatment Regimens

Treatment regimens were determined by the treating physicians according to standard clinical practice and were not influenced by the study protocol.

- Before switching: SHL rFVIII concentrates;
- After switching: efmoroctocog alfa.

Dosing, infusion frequency, and regimen adjustments were individualized based on clinical phenotype, bleeding frequency, physician judgment and patient adherence and lifestyle.

### 2.4. Data Collection

Data were collected at three predefined time points: (i) Baseline (E1, at treatment switch), (ii) 6 months (E2 ± 1 month) and (iii) 12 months (E3 ± 1 month).

Data sources included medical records, patient diaries and PROs.

### 2.5. Study Variables

The following variables were collected:

- Demographic and clinical characteristics: age, age at diagnosis and disease severity.
- Bleeding outcomes: annualized bleeding rate (ABR), annualized joint bleeding rate (AjBR) and number and type of bleeding episodes.
- Joint health: Hemophilia Joint Health Score (HJHS), assessed by trained clinicians.
- Pain assessment: Pain was assessed using the visual analog scale (VAS) ranging from 0 (no pain) to 10 (worst imaginable pain).
- Treatment parameters: Infusion frequency (infusions per week) and FVIII consumption (IU/kg/year).
- Healthcare utilization: Number and cause of hospitalizations.

### 2.6. Outcome Measures

Primary outcomes were assessed by change in ABR.

Secondary outcomes were assessed by change in AjBR, HJHS, pain score, infusion frequency, FVIII consumption and hospitalization.

Exploratory outcomes included subgroup analyses by age (<22 vs. ≥22 years) and subgroup analyses based on pre-switch treatment (on-demand vs. prophylaxis).

Analyses were conducted using available data without imputation. The number of patients contributing to each endpoint varied depending on data availability. For HJHS and pain assessments, analyses were based on smaller subsets of patients with available paired data.

HJHS assessments were performed by trained clinicians at each participating center according to standard clinical practice. Formal central standardization was not implemented.

### 2.7. Safety Assessment

Safety evaluation included: recording of adverse events (AEs) and monitoring for development of FVIII inhibitors. Inhibitor testing was performed according to local clinical practice.

### 2.8. Statistical Analysis

Descriptive statistics were used to summarize baseline characteristics and outcomes:

- Continuous variables: mean ± standard deviation (SD);
- Categorical variables: frequencies and percentages.

Comparisons between pre- and post-switch data were performed using:

- Paired *t*-tests for normally distributed variables;
- Wilcoxon signed-rank tests for non-normally distributed variables;
- Chi-square or Fisher’s exact tests for categorical variables.

A *p*-value < 0.05 was considered statistically significant.

To enhance interpretability, changes were also presented as absolute differences and percentage reductions; subgroup analyses were conducted where appropriate.

Missing data were not imputed; analyses were based on available data.

Given the relatively small sample size and heterogeneity of the cohort, no adjustment for multiplicity was performed, and findings should be interpreted as exploratory.

### 2.9. Ethical Considerations

The study was conducted in accordance with the World Medical Association Declaration of Helsinki and the International Council for Harmonization Good Clinical Practice guidelines.

Ethical approval was obtained from the relevant institutional review boards of all participating centers.

All participants (or legal guardians) provided written informed consent prior to inclusion.

## 3. Results

### 3.1. Demographics

Sixty male Caucasian patients participated: 51 (85%) with severe and nine (15%) with moderate HA, mean age 27.9 ± 15.3 years, and mean diagnosis age 2.3 ± 5.0 years. Nineteen patients were <19 years old (32%), 27 between 19 and 40 years of age (45%) and 14 patients more than 40 years old (23%).

*Treatment*

All participants received HA treatment in the preceding 12 months before starting efmoroctocog alfa: 16 (26.7%) on-demand and 44 (73.3%) prophylactically. All patients treated on-demand were followed at the adult hemophilia centers; prophylaxis had not been introduced in these patients due to difficulties in venous access in the past and non-severe bleeding phenotype. Regarding the patients treated prophylactically, they were divided in two groups: (i) adults with severe hemophilia A and severe bleeding phenotype (n = 23) and (ii) all children and adolescents (n = 19) included in the study; they had all been on early prophylaxis before two years of age or after the first joint bleeding.

Six SHL FVIII concentrates were administered, with treatment duration ranging from <5 to >11 years. Dosage analysis pre- and post-efmoroctocog alfa initiation is detailed in Table 1.

### 3.2. Bleeding Episodes

Patient numbers and ABR/patient pre-switch (E1) and post-switch (E2/E3) are shown in Table 2. ABR significantly decreased from 6.8 to 3.2 after the switch (*p* < 0.001).

Notably, during the year preceding the switch, 44 (73.3%) patients received prophylaxis; in follow-up periods, all but one were on prophylactic regimens.

Six (10%) patients required hospitalization pre-switch—two for knee arthroplasty, four for severe bleeding. In each consecutive 6-month period post-switch, one patient (1.7%) required hospitalization (myringotomy and transtrochanteric fracture).

**Figure 1:**
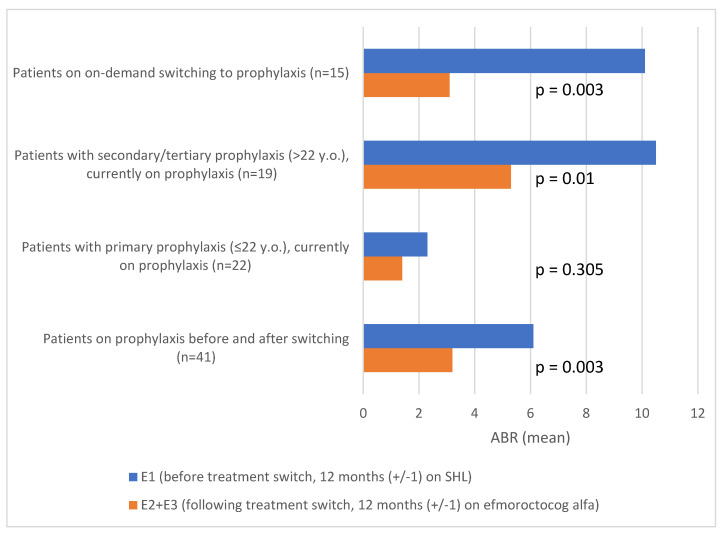
Annualized bleeding rate (ABR) 12 months prior and 12 months after efmoroctocog alfa initiation of patients on prophylactic treatment.

For patients on prophylaxis with available data, ABR/patient declined from 6.1 pre-switch to 3.2 post-switch (*p* = 0.003; Figure 1).

Age-group analysis revealed no significant difference in ABR for patients < 22 years comparing E1 and E2/E3 (2.3 vs. 1.4, *p* = 0.305); however, older patients (≥22 years) exhibited significant reduction (10.5 vs. 5.3, *p* = 0.01; Figure 1).

Switching from on-demand SHL rFVIII treatment to rFVIIIFc prophylaxis was associated with a reduction in ABR/patient from 10.1 to 3.1 (*p* = 0.003; Figure 1).

It should be taken into consideration that reductions in ABRs were more pronounced among patients who transitioned from on-demand therapy to prophylaxis, whereas more modest improvements were observed among patients already receiving prophylaxis prior to switching.

### 3.3. Joint Bleeding Episodes

Comparison of joint bleeding episodes over two consecutive 12-month periods showed that 39 patients reported such episodes pre-switch, while 26 did so post-switch, yielding a significant decrease in total AjBR and AjBR/patient (*p* < 0.001; Table 3).

Among those on prophylaxis, findings were consistent: no significant change for patients < 22 years, but substantial reduction for patients ≥ 22 years (*p* = 0.002; Table 4).

Transition from on-demand to prophylactic rFVIIIFc resulted in lower AjBR (9.9 vs. 3.5, *p* < 0.001).

### 3.4. Joint Health

**Figure 2:**
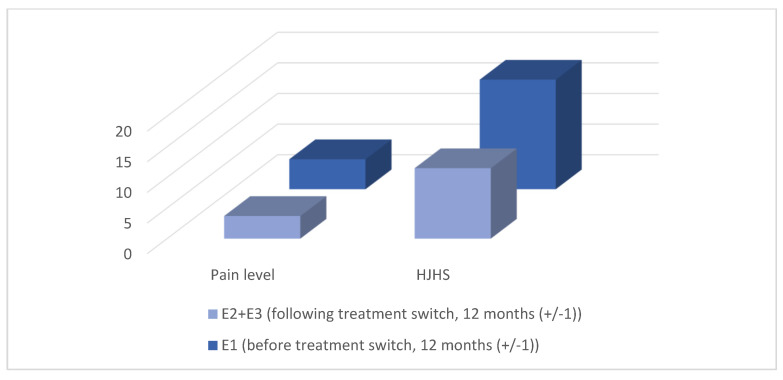
Hemophilia Joint Health Score (HJHS) measurements and pain levels (measured on a visual analog scale) 12 months prior and 12 months following efmoroctocog alfa initiation.

HJHS reflected the following improvements: Among patients with available paired assessments, mean HJHS decreased from 17.9 ± 21.3 at baseline to 11.5 ± 15.5 during follow-up (*p* < 0.007; Figure 2).

### 3.5. Pain Level

Seventeen patients had mean pain scores of 4.9 ± 1.4 at E1. At E2, mean scores decreased to 3.8 ± 1.8. Over the 12 months post-switch (E2/E3), 18 patients averaged 3.7 ± 1.7, a significant improvement (*p* < 0.001; Figure 2).

It should be noted that HJHS and pain outcomes were evaluated in smaller subsets of patients with available paired data and should therefore be interpreted with caution.

### 3.6. FVIII Consumption

Data on FVIII concentrate consumption (in IU) during the 12 months prior to rFVIIIFc initiation (E1) and 12 months following switch (E2 and E3) were compared. Data for all patients receiving prophylactic treatment were used for the analysis (Table 5).

For the PWHA on prophylaxis prior to and after the switch, weekly infusion frequency decreased from 3.2 to 2.2 (*p* < 0.001), while mean dose per infusion increased from 27.4 to 38.1 IU/kg (*p* < 0.001). As a result, total annual FVIII consumption did not consistently decrease across the cohort (Table 5). These findings suggest that the primary benefit relates to reduced infusion burden rather than reduced overall factor utilization.

### 3.7. Safety

No treatment-related adverse events were reported following treatment switch to rFVIIIFc. No patient developed inhibitors while on treatment with rFVIIIFc.

## 4. Discussion

This multicenter, non-interventional study evaluated the impact of switching moderate to severe HA patients from SHL FVIII (prophylactic/on-demand) to efmoroctocog alfa in real-world clinical settings, analyzing both clinical outcomes and PROs over one year before and after transition.

It is worth noting that, in the Greek hemophilia care setting, treatment patterns have historically differed between adults and children. Many adults with hemophilia remained on on-demand treatment, partly because regular prophylaxis with standard half-life FVIII required frequent intravenous infusions, which could pose practical difficulties and affect the uptake of long-term prophylactic regimens. In contrast, children in Greece have generally been introduced to early prophylaxis since 2000, typically initiated before 2 years of age and before the development of significant joint disease, reflecting a gradual shift toward preventive management in the pediatric population [15]. These historical differences may also help explain, at least in part, the variation in the pre- and post-switch patient sample with regard to the proportion of patients receiving prophylaxis. In particular, the lower proportion of patients on prophylaxis before switching may reflect the inclusion of older adult patients who had remained on on-demand treatment for many years, whereas after switching most patients were managed with prophylaxis. Accordingly, the observed changes should be interpreted in the context of both product switching and the broader evolution of treatment practice in Greece.

Results demonstrate that efmoroctocog alfa prophylaxis was associated with improvements in key clinical outcome measures for HA, including ABR, AjBR, HJHS, and pain level, with weekly infusion frequency either comparable or statistically improved across all age categories. The pre-post observational design introduces several potential sources of bias. Regression to the mean may partially explain reductions in bleeding rates, particularly in patients switched due to higher baseline bleeding frequency. In addition, improved adherence, increased clinical monitoring, and lifestyle modifications following treatment switch might have enhanced the observed outcomes. Selection bias cannot be excluded, as patients selected for switching may differ from those remaining on prior treatment. Nevertheless, our findings align with previous studies showing equal or improved ABRs for efmoroctocog alfa compared to SHL FVIII products, along with enhanced adherence, HRQoL, and bleed protection [4,7,16,17,18].

Limitations include the small sample size (60 participants, with fewer completing E2/E3 visits), which may limit generalizability, as well as the short follow-up, cohort heterogeneity, and missing data for some outcomes (e.g., HJHS and pain). In addition, the absence of a control group limits causal interpretation.

Safety findings should also be interpreted cautiously, as the relatively small cohort size and limited follow-up duration reduce the ability of the study to detect uncommon adverse events or inhibitor development.

Despite these limitations, this study contributes valuable evidence regarding the efficacy and benefits of efmoroctocog alfa in moderate to severe HA. The observed reductions in bleeding rates, joint bleeding episodes, and pain, together with improvements in joint health and reduced infusion frequency, support the routine use of efmoroctocog alfa as an effective prophylactic option in both adult and pediatric patients with moderate to severe hemophilia A.

Importantly, the reduction in treatment burden—reflected by fewer weekly infusions—may improve patient adherence, which remains a critical determinant of long-term outcomes in hemophilia care. The ability to maintain higher FVIII trough levels with less frequent dosing may also enable patients to engage more safely in physical activity, thereby contributing to improved overall quality of life.

From a healthcare system perspective, the reduction in bleeding episodes and hospitalizations suggests a potential decrease in resource utilization and associated costs. These findings reinforce the value of transitioning suitable patients from on-demand or standard half-life therapies to extended half-life prophylaxis in real-world clinical settings.

The evolving therapeutic landscape of hemophilia A includes non-factor therapies such as emicizumab, which have demonstrated substantial reductions in bleeding rates with subcutaneous administration. In this context, EHL FVIII products may still play an important role, particularly in patients requiring flexible dosing, perioperative management, or individualized pharmacokinetic-guided prophylaxis.

Future studies should explore comparative effectiveness between extended half-life products and emerging non-factor therapies, as well as potential combination or sequential treatment strategies. Comparative head-to-head real-world studies are needed to better define the positioning of these treatment options.

In conclusion, this real-world study suggests that switching to efmoroctocog alfa was associated with improvements in bleeding outcomes, joint-related parameters, and infusion frequency. However, given the observational design and the heterogeneity of the study population, these findings should be interpreted with appropriate caution as real-world observations rather than confirmatory evidence.

## Figures and Tables

**Table 1:** Descriptive analysis of FVIII concentrates dosage for the period up to 12 months (±1 month) prior to efmoroctocog alfa initiation (visit E1) and 12 months (±1 month) following efmoroctocog alfa initiation (visits Ε2 and Ε3) for all patients for whom infusion data were available.

**VISIT E1-BASELINE**		
Type of treatment	**Prophylaxis**	**On-demand**
Number of patients	38	15
	Median (range)	
Infusions/week (n)	3.1 (0.7–7.7)	0.6 (0.1–2.1)
Dose per infusion (IU/kg)	26.6 (11.2–40.7)	29.1 (16.7–42.5)
Total factor consumption (IU)	199,310 (30,390–905,030)	83,982 (9998–205,949)
Weekly infusion dose (IU/kg)	67.2 (18.4–206.7)	-
**VISITS E2/E3**		
	**All-patients**	**Patients on Prophylaxis**
Number of patients	53	50
	Median (range)	
Infusions/week (n)	2.2 (0.8–6.9)	2.2 (0.9–6.9)
Dose per infusion (IU/kg)	35.9 (22.3–65.3)	36.0 (22.3–65.3)
Total factor consumption (IU)	223,084 (67,410–981,598)	221,533 (67,410–981.598)
Weekly infusion dose (IU/kg)	-	70.0 (22.6–224.2)

*IU: International Unit.*

**Table 2:** Patients with bleeding episodes and ABR/patient prior (Visit E1) and after (Visits E2 and E3) efmoroctocog alfa initiation.

Visit	Patients with Bleeding Episodes, n (%)	ABR ^†^ per Patient
E1	40 (66.7)	6.8
E2	26 (44.8)	4.1
E3	21 (35.0)	2.4
E2/E3	29 (48.3)	3.2

*^†^ ABR: Annualized Bleeding Rate.*

**Table 3:** Joint bleeding rate prior and following switch to efmoroctocog alfa.

Period	12 Months Before Switch	12 Months Following Switch	*p*-Value
Number of patients	60	58	
Total AjBR ^†^	385	168	-
AjBR ^†^/patient (Mean ± SD ^‡^)	6.4 (9.2)	2.9 (6.1)	<0.001
AjBR ^†^/patient (Median, Min; Max)	2 (0; 40)	0 (0; 34)	<0.001

*^†^ AjBR: annualized joint bleeding rate; ^‡^ SD: standard deviation.*

**Table 4:** Joint bleeding rate prior and following switch to efmoroctocog alfa in patients who were and continued being on prophylaxis.

**Age group < 22 years**			
Type of treatment	**12 months** **before switch**	**12 months following switch**	***p*-value**
Number of patients	22	22	
Total AjBR ^†^	38	26	-
AjBR ^†^/patient (Mean ± SD ^‡^)	1.7 (2.9)	1.2 (2.5)	0.442
AjBR ^†^/patient (Median, Min; Max)	0 (0; 8)	0 (0; 11)	0.442
**Age group ≥ 22 years**			
Type of treatment	**12 months** **before switch**	**12 months following switch**	***p*-value**
Number of patients	19	19	
Total AjBR ^†^	191	89	-
AjBR ^†^/patient (Mean ± SD ^‡^)	10.1 (13.3)	4.7 (8.8)	0.002
AjBR ^†^/patient (Median, Min; Max)	4 (0; 40)	1 (0; 34)	0.002

*^†^ AjBR: annualized joint bleeding rate; ^‡^ SD: standard deviation.*

**Table 5:** Comparison of infusions between Ε1 and E2/Ε3 for all the patients on prophylactic treatment.

Measure	Before Switch	After Switch	*p*-Value
Weekly infusions	3.1	2.2	<0.001
Infusion dose (IU/kg)	26.6	36.0	<0.001
Total FVIII consumption (IU)	199,310	221,533	<0.001

*IU: International Unit.*

## Data Availability

The original contributions presented in this study are included in the article. Further inquiries can be directed to the corresponding author.

## References

[1] Varaklioti A., Kontodimopoulos N., Niakas D., Kouramba A., Katsarou O. (2017). Health-Related Quality of Life and Association With Arthropathy in Greek Patients with Haemophilia. Clin. Appl. Thromb. Haemost..

[2] Srivastava A., Brewer A.K., Mauser-Bunschoten E.P., Key N.S., Kitchen S., Llinas A., Ludlam C.A., Mahlangu J.N., Mulder K., Poon M.C. (2013). Treatment Guidelines Working Group on Behalf of The World Federation of Hemophilia. Guidelines for the management of haemophilia. Haemophilia.

[3] White G.C., Rosendaal F., Aledort L.M., Lusher J.M., Rothschild C., Ingerslev J., Factor VIII and Factor IX Subcommittee (2001). Definitions in haemophilia. Recommendation of the scientific subcommittee on factor VIII and factor IX of the scientific and standardization committee of the International Society on Thrombosis and Haemostasis. Thromb. Haemost..

[4] Mahlangu J., Powell J.S., Ragni M.V., Chowdary P., Josephson N.C., Pabinger I., Hanabusa H., Gupta N., Kulkarni R., Fogarty P. (2014). Phase 3 study of recombinant factor VIII Fc fusion protein in severe haemophilia A. Blood.

[5] Berntorp E., Spotts G., Patrone L., Ewenstein B.M. (2014). Advancing personalized care in haemophilia A: Ten years’ experience with an advanced category antihemophilic factor prepared using a plasma/albumin-free method. Biologics.

[6] Fischer K., van der Bom J.G., Molho P., Negrier C., Mauser-Bunschoten E.P., Roosendaal G., De Kleijn P., Grobbee D.E., Berg H.M.V.D. (2002). Prophylactic versus on-demand treatment strategies for severe haemophilia: A comparison of costs and long-term outcome. Haemophilia.

[7] Wang C., Young G. (2018). Clinical use of recombinant factor VIII Fc and recombinant factor IX Fc in patients with haemophilia A and B. Haemophilia.

[8] Graf L. (2018). Extended Half-Life Factor VIII and Factor IX Preparations. Transfus. Med. Hemotherapy.

[9] Lambert T., Benson G., Dolan G., Hermans C., Jiménez-Yuste V., Ljung R., Morfini M., Zupančić-Šalek S., Santagostino E. (2018). Practical aspects of extended half-life products for the treatment of haemophilia. Ther. Adv. Hematol..

[10] McMullen S., Buckley B., Hall E., Kendter J., Johnston K. (2017). Budget Impact Analysis of Prolonged Half-Life Recombinant FVIII Therapy for Haemophilia in the United States. Value Health.

[11] Henry N., Jovanović J., Schlueter M., Kritikou P., Wilson K., Myrén K.J. (2017). Cost-utility analysis of life-long prophylaxis with recombinant factor VIIIFc vs recombinant factor VIII for the management of severe haemophilia A in Sweden. J. Med. Econ..

[12] Peyvandi F., Garagiola I. (2015). Treatment of haemophilia in the near future. Semin. Thromb. Haemost..

[13] Nolan B., Mahlangu J., Perry D., Young G., Liesner R., Konkle B., Rangarajan S., Brown S., Hanabusa H., Pasi K.J. (2016). Long-term safety and efficacy of recombinant factor VIII Fc fusion protein (rFVIIIFc) in subjects with haemophilia A. Haemophilia.

[14] Jiménez-Yuste V., Auerswald G., Benson G., Lambert T., Morfini M., Remor E., Salek S.Z. (2014). Achieving and maintaining an optimal trough level for prophylaxis in haemophilia: The past, the present and the future. Blood Transfus..

[15] Michalopoulou A., Ranta S., Andersson N.G., Fischer K., de Kovel M., de Boer-Verdonk E., Motwani J., Kenet G., Pergantou H. (2025). Differences Between Sweden and Greece in Joint Outcomes Assessed by Ultrasound in Adolescents With Severe Haemophilia on Prophylaxis: Data From the PedNet Registry. Haemophilia.

[16] Young G., Mahlangu J., Kulkarni R., Nolan B., Liesner R., Pasi J., Barnes C., Neelakantan S., Gambino G., Cristiano L.M. (2015). Recombinant factor VIII Fc fusion protein for the prevention and treatment of bleeding in children with severe haemophilia A. J. Thromb. Haemost..

[17] Varaklioti A., Kontodimopoulos N., Katsarou O., Niakas D. (2014). Psychometric properties of the Greek Haem-A-QoL for measuring quality of life in Greek haemophilia patients. BioMed Res. Int..

[18] Dettoraki A., Michalopoulou A., Mazarakis M., Saslis S., Stamati I., Kapsimali Z., Pergantou H. (2022). Clinical application of extended half-life factor VIII in children with severe haemophilia A. Haemophilia.

